# Reduction-Responsive Boc-Modified Gelatin-Based Hydrogels for Enhanced Hydrophobic Drug Loading and Controlled Release

**DOI:** 10.3390/gels12070614

**Published:** 2026-07-09

**Authors:** Shuo Wang, Ruxin Zhang, Xiangyu Chen, Helong Wang, Yingfei Hu, Yuanxi Zhu, Wenhui Lu, Deyi Zhu

**Affiliations:** 1 State Key Laboratory of Green Papermaking and Resource Recycling, Faculty of Light Industry, Qilu University of Technology (Shandong Academy of Sciences), Jinan 250353, China; 2 Bloomage Biotech Research and Development Center for Zero-Carbon Generic Technologies in the Biomedical Industry, Qilu University of Technology (Shandong Academy of Sciences), Jinan 250353, China

**Keywords:** gelatin-based hydrogel, Boc modification, reduction-responsive, drug controlled release, hydrophobic drug delivery

## Abstract

Conventional gelatin-based hydrogels lack responsiveness to the tumor microenvironment and exhibit low drug-loading efficiency for hydrophobic drugs, which limits their application in targeted cancer therapy. In this study, a reduction-responsive gelatin hydrogel was developed by grafting hydrophobic tert-butoxycarbonyl (Boc) groups onto gelatin chains, followed by EDC/NHS-mediated chemical cross-linking with L-cysteine dimethyl ester dihydrochloride, which contains disulfide bonds. The success of Boc grafting was confirmed by ^1^H NMR, and free amine quantitative analysis. Rheological characterization confirmed the formation of a stable elastic network with controllable gelation times (5–12 min), demonstrating excellent injectability. Reduction-triggered degradation was observed in the presence of DTT or GSH, with the cleavage of disulfide bonds further evidenced by the detection of free sulfhydryl (-SH) groups via X-ray Photoelectron Spectroscopy. Microscale thermophoresis (MST) demonstrated measurable binding between Boc-modified gelatin and hydrophobic drugs, with dissociation constants (K_d_) of 0.46 ± 0.37 μM for curcumin and 0.14 ± 0.14 μM for camptothecin. Drug loading assays show dramatically enhanced encapsulation efficiency for hydrophobic drugs (62.7% for curcumin, 66.6% for camptothecin) compared to unmodified hydrogels (7.2% and 16.8%, respectively). In vitro release kinetics follow the Korsmeyer–Peppas model, indicating a non-Fickian diffusion–erosion mechanism, and can be precisely tuned by gelatin concentration and reducing agent levels. Complete drug release occurs within approximately 325 min in 15 mM GSH. MTT and hemolysis assays confirm high biocompatibility. Collectively, this reduction-responsive system offers a promising platform for controlled, site-specific delivery of hydrophobic anticancer agents.

## 1. Introduction

Cancer remains one of the leading causes of death worldwide. Conventional systemic chemotherapy is often hindered by low drug bioavailability, severe off-target toxicity, and insufficient tumor specificity. These limitations not only reduce therapeutic efficacy but also result in significant systemic side effects [1,2]. Consequently, increasing attention has been directed toward the development of safer and more effective targeted drug delivery systems capable of achieving high drug-loading efficiency and precise, site-specific release [3]. Among the various carriers, hydrogel materials characterized by high water content and a three-dimensional polymer network structure have emerged as highly promising candidates [4]. However, traditional hydrogels often lack the ability to respond to the pathological microenvironment, leading to uncontrolled drug release and thereby limiting their therapeutic efficacy [5].

Gelatin, a natural biopolymer derived from collagen, has been widely explored in biomedical applications due to its inherent biocompatibility, biodegradability and ease of processing under mild conditions [6,7]. Moreover, gelatin molecular chains contain a wealth of reactive functional groups, including the ε-amino group of lysine residues and the carboxyl groups of glutamic and aspartic acid residues [8,9]. These functional groups provide ideal reaction sites for the chemical modification of gelatin and have been widely applied in the fields of tissue engineering, wound dressings, and drug delivery [10,11].

Despite these advantages, conventional gelatin-based hydrogels exhibit poor responsiveness to the tumor microenvironment, particularly showing insufficient sensitivity to the elevated glutathione (GSH) levels in cancer cells [12,13]. The intracellular GSH concentration in tumor cells (2–10 mM) is significantly higher than that in normal cells and the extracellular environment (2–20 µM), driven primarily by elevated oxidative stress and enhanced GSH synthesis associated with cancer metabolism and chemoresistance [14]. This stark concentration gradient not only contributes to the resistance of cancer cells to certain chemotherapeutics but, conversely, provides a unique biochemical cue for designing smart drug delivery systems. Reduction-responsive hydrogels have been developed by introducing disulfide bonds (-S-S-) into the polymer network. These bonds remain stable under normal physiological conditions, but are cleaved in the reducing intracellular environment of tumors, leading to network degradation and triggered drug release. This strategy has been successfully applied to various polymer systems [15,16]. Another critical challenge for gelatin-based hydrogels is their limited capacity to load hydrophobic drugs. As a predominantly hydrophilic protein, gelatin lacks sufficient hydrophobic domains to effectively interact with hydrophobic drug molecules, leading to poor drug-loading efficiency. This is particularly problematic since many anticancer drugs are hydrophobic in nature [17].

**Figure 1:**
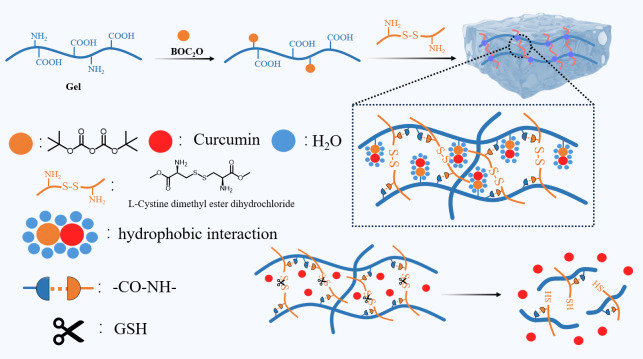
Schematic illustration of design strategies for the reductive-responsive gelatin-based hydrogels.

In this study, we designed a reduction-responsive gelatin-based hydrogel with enhanced hydrophobic drug-loading capability. As illustrated in Figure 1, hydrophobic tert-butyloxycarbonyl (Boc) groups were first grafted onto gelatin chains via modification with di-tert-butyl dicarbonate, introducing hydrophobic domains for improved drug affinity. Subsequently, the Boc-modified gelatin (Gel@Boc) was chemically cross-linked with L-cysteine dimethyl ester dihydrochloride using an *N*-(3-Dimethylaminopropyl)-*N′*-ethylcarbodiimide hydrochloride (EDC·HCl) and *N*-Hydroxysuccinimide (NHS)-mediated coupling strategy, forming a stable three-dimensional network containing disulfide linkages. The incorporation of Boc groups and methyl ester groups enhances interactions with hydrophobic drugs, while the disulfide-crosslinked structure imparts reduction-responsive degradability, enabling controlled drug release in reductive environments.

## 2. Results and Discussion

### 2.1. Characterization of Gel@Boc and Gel@Boc-Dc Hydrogels

Di-tert-butyl dicarbonate (Boc_2_O) is widely used to introduce the tert-butoxycarbonyl (Boc) protecting group, selectively masking primary and secondary amines [18]. The successful grafting of Boc groups onto gelatin was confirmed through spectroscopic and chemical analyses. In the ^1^H NMR spectrum of Gel@Boc, a characteristic intensification of the resonance at δ 1.26 ppm was observed, which is characteristic of the methyl hydrogen protons of the tert-butyl group in the Boc moiety [19]. Notably, while unmodified gelatin exhibits a weak signal at the same chemical shift due to the overlapping methyl resonances of hydrophobic amino acid residues (e.g., alanine, valine, leucine, and isoleucine) [20], the marked increase in signal intensity in the Gel@Boc sample confirms the successful grafting of Boc groups. Furthermore, the resonance corresponding to the methylene protons adjacent to the ε-amino groups of lysine residues underwent a downfield shift from 2.84 ppm to 2.90 ppm [20]. This shift reflects the change in the electronic environment resulting from the transformation of free amines into carbamate moieties, providing robust evidence for the covalent conjugation of Boc groups to the gelatin backbone [21].

**Figure 2:**
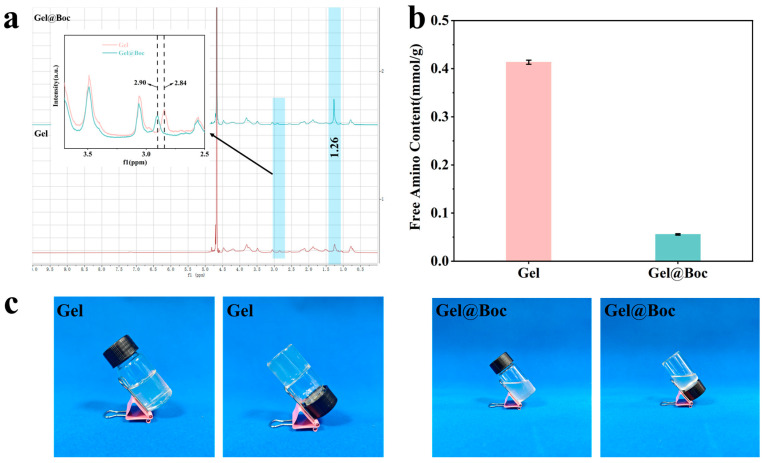
Structural characterization of Gel@Boc: (**a**) ^1^H NMR spectra of gelatin and Gel@Boc in D_2_O, confirming successful Boc grafting; (**b**) quantitative results of free amino group content before and after Boc modification, determined using the OPA method, confirming the successful Boc grafting and the masking of amino groups; (**c**) photographs of gelatin (5 wt%) and Gel@Boc (5 wt%) solutions after standing at room temperature for 1 h.

Gelatin contains a certain amount of free amino groups, predominantly originating from the ε-amino groups of lysine residues [22]. To optimize Boc modification, gelatin was reacted with varying Boc concentrations, and the residual free amino group content was quantified using the o-phthalaldehyde (OPA) assay (Figure S1). Based on these results, a Boc concentration that produced near-maximal grafting was selected for subsequent experiments. Quantitative OPA analysis at this concentration (Figure 2b) indicated a Boc grafting efficiency of approximately 87%, confirming effective masking of free amino groups through Boc functionalization. Collectively, these results demonstrate successful hydrophobic functionalization of gelatin, introducing domains capable of interacting with hydrophobic molecules.

Boc modification substantially alters the intrinsic sol–gel transition behavior of gelatin. In contrast to native gelatin, which undergoes thermoreversible gelation through triple helix formation upon cooling [23], Gel@Boc remains in a sol state at room temperature (Figure 2c). This effect is attributed to steric hindrance imposed by bulky tert-butyl groups and disruption of hydrogen bonding, which inhibit chain alignment and helix formation [24]. This observation not only further confirms the successful incorporation of Boc moieties but also demonstrates that Gel@Boc is incapable of forming a physically crosslinked gel network through intrinsic gelatin interactions.

### 2.2. Gelation Behavior of Gel@Boc-Dc Hydrogels

**Figure 3:**
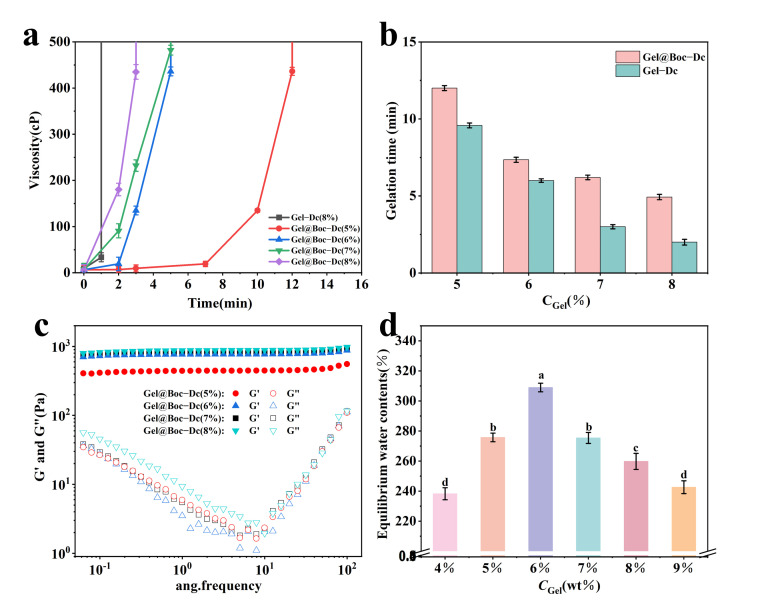
Gelation behavior, rheological properties and swelling behavior of Gel@Boc-Dc hydrogels: (**a**) time-dependent viscosity evolution during the Sol–Gel transformation of Gel@Boc-Dc; (**b**) gelation time as a function of gelatin concentration; (**c**) frequency-dependent storage modulus (G′) and loss modulus (G″) of Gel@Boc-Dc hydrogels; (**d**) swelling behavior of Gel@Boc-Dc hydrogels with varying gelatin concentrations. Different lowercase letters in the figure indicate statistically significant differences between groups (*p* < 0.05, *n* = 3).

Upon EDC/NHS-mediated crosslinking with L-cysteine dimethyl ester, Gel@Boc-Dc hydrogels are formed via covalent amide bond formation, establishing a chemically crosslinked three-dimensional network. Compared with native gelatin, Gel@Boc-Dc exhibits a markedly slower viscosity evolution during gelation (Figure 3a), which can be attributed to the substantial reduction in available nucleophilic amine groups following Boc protection. This limitation in reactive sites decreases crosslinking probability and delays network percolation, thereby moderating the gelation process.

Consistent with this mechanism, gelation time decreases when polymer concentration increases (Figure 3b), since higher chain density promotes intermolecular collision frequency and thereby expedites network formation. However, even at elevated concentrations, Gel@Boc-Dc retains longer gelation times than unmodified gelatin, highlighting the dominant role of chemical modification in regulating gelation kinetics. Importantly, the gelation window (5–12 min) falls within an optimal range for injectable systems, enabling sufficient processing time while ensuring rapid in situ stabilization post-administration [25].

Rheological analysis (Figure 3c) further elucidates the network characteristics. The consistent dominance of the storage modulus (G′) over the loss modulus (G″), coupled with weak frequency dependence, indicates the formation of a well-developed, elastically percolated network with stable crosslinking points. This behavior suggests that chemical crosslinks, rather than physical entanglements, govern the mechanical integrity of the hydrogel [26]. The loss modulus (G″) exhibits a characteristic V-shaped dependence on angular frequency, reflecting the mechanically stable hydrogel networks. In the low-frequency region, G″ progressively decreased with increasing frequency, which can be attributed to the sufficient relaxation of polymer chains and solvent-associated structures during each oscillation cycle, resulting in reduced viscous dissipation [27]. As the frequency approached the intermediate regime, G″ reached a minimum, corresponding to a characteristic relaxation frequency of the hydrogel network. Conversely, in the high-frequency region, the rapid oscillation outpaces the characteristic relaxation time of the network, where restricted chain mobility and localized deformations enhance energy dissipation, resulting in a significant rise in G″ [28].

The swelling behavior (Figure 3d) provides additional insight into the internal network architecture. When the gelatin concentration exceeds 6 wt%, increasing polymer content results in reduced equilibrium swelling, reflecting higher crosslinking density and a corresponding reduction in network mesh size. This densification restricts solvent diffusion and thereby limits water uptake [29]. It should be noted that, at lower concentrations, insufficient chain entanglement and crosslinking lead to heterogeneous and loosely connected networks, which are prone to partial disintegration during swelling, yielding reduced apparent swelling ratios [30]. Such structure-swelling relationships have been widely reported in hydrogel systems, where mesh size and crosslink density serve as key determinants of water absorption and transport behavior [31].

Collectively, these results demonstrate a strongly interdependent relationship between chemical modification, crosslinking kinetics, and network architecture. Through modulation of polymer concentration and Boc functionalization, both gelation behavior and structural properties of the hydrogel can be controlled, providing a tunable synthesis strategy for optimizing drug delivery functionality.

### 2.3. Micromorphology of Gel@Boc-Dc Hydrogels

**Figure 4:**
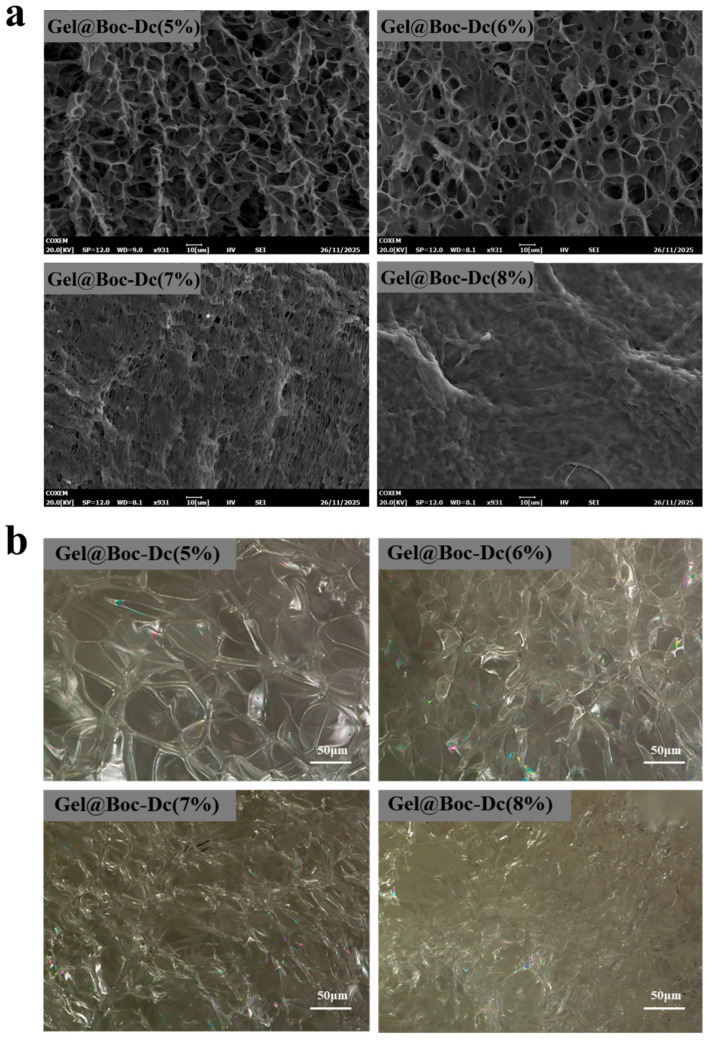
Microstructure of Gel@Boc-Dc hydrogels with varying gelatin concentrations: (**a**) SEM images; (**b**) optical microscopy images obtained with SDFM.

Figure 4 presents the micromorphological characterization of freeze-dried Gel@Boc-Dc hydrogels using digital microscopy and scanning electron microscopy (SEM). All samples exhibited a typical three-dimensional porous network structure, which provides ample space for drug encapsulation and ensures homogeneous dispersion of therapeutic agents within the gel matrix. At low gelatin concentrations, the hydrogel surface displayed high porosity. As the gelatin concentration increased, the pore structure became more compact and the number of pores substantially decreased.

The variation in pore architecture significantly influences drug loading and release behavior, including drug loading capacity, encapsulation efficiency, and release kinetics. Generally, larger pore sizes and higher porosity facilitate rapid drug diffusion and promote drug release from the hydrogel interior. In contrast, denser networks with lower porosity prolong drug diffusion pathways, slow down the release rate, and mitigate burst release, thereby achieving sustained release. Notably, microscale pore structures (with pore sizes in the range of 1–100 µm) offer unique advantages in applications such as tissue engineering scaffolds, macromolecular drug delivery, and cell culture. For instance, Kim Sungjun et al. [32] reported porous structures with pore sizes ranging from 20 to 80 µm achieved by tuning polymer concentration and crosslinking conditions. Such structures significantly improved the swelling capacity and mechanical elasticity of the hydrogels while providing ample space for the encapsulation of macromolecular drugs or growth factors.

### 2.4. Reduction-Responsiveness

**Figure 5:**
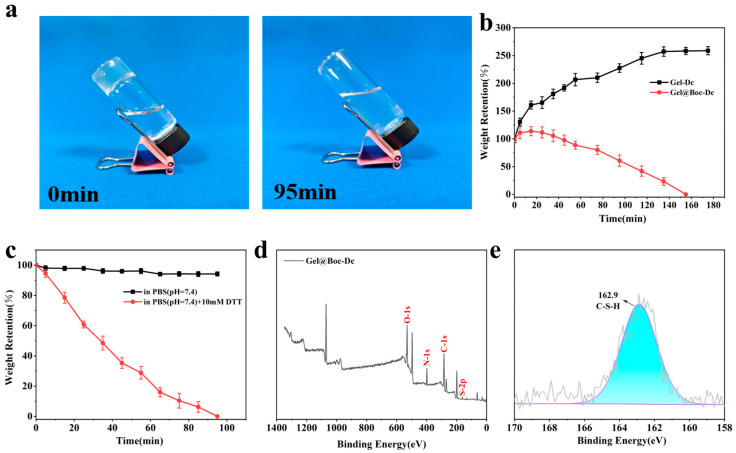
Reduction-responsive degradation of Gel@Boc-Dc (6%) hydrogels: (**a**) macroscopic degradation process in DTT solution; (**b**) water absorption–disintegration curves of Gel-Dc (6%) and Gel@Boc-Dc (6%) hydrogels in 10 mM DTT; (**c**) mass changes in Gel@Boc-Dc (6%) hydrogels in PBS and DTT-containing media; (**d**) XPS total spectra of degradation products from the aforementioned Gel@Boc-Dc (6%) hydrogels in DTT-containing media; (**e**) high-resolution S2p spectra confirming the formation of thiol groups following disulfide bond cleavage.

The redox-responsive degradation behavior of Gel@Boc-Dc (6%) hydrogels was evaluated using dithiothreitol (DTT) as a reducing agent. As shown in Figure 5a, the hydrogel underwent rapid structural disintegration, ultimately forming a homogeneous solution within 95 min, indicating efficient network cleavage under reductive conditions. The swelling–degradation profiles (Figure 5b) reveal a distinct two-stage behavior. In the initial stage, both Gel-Dc (6%) and Gel@Boc-Dc (6%) hydrogels exhibited a mass increase due to rapid water uptake and swelling. However, after approximately 20 min, the mass of Gel@Boc-Dc (6%) decreased continuously, reflecting progressive degradation of the network. This transition arises from reductive cleavage of disulfide crosslinks, which reduces crosslinking density and ultimately disrupts network connectivity, leading to macroscopic dissolution. In contrast, the hydrogel derived from native gelatin (Gel-Dc) showed no mass loss under the same conditions and instead reaches swelling equilibrium, indicating the absence of reduction-sensitive linkages. This difference underscores that Gel-Dc (6%) primarily relies on intrinsic gelatin interactions for network formation, whereas Boc modification suppresses such physical crosslinking by masking amino groups. The subsequent incorporation of disulfide bonds therefore serves as the dominant crosslinking mechanism in Gel@Boc-Dc (6%), conferring redox responsiveness to the hydrogel system. Further validation is provided by degradation studies in different media (Figure 5c). The hydrogel remains structurally stable in PBS (pH 7.4), exhibiting negligible mass change, whereas continuous mass loss is observed in PBS containing 10 mM DTT until complete degradation. This marked contrast confirms that hydrogel disintegration is specifically triggered by reductive cleavage of disulfide bonds rather than nonspecific hydrolysis.

To elucidate the molecular mechanism, degradation products were analyzed by X-ray photoelectron spectroscopy (XPS). The survey spectrum (Figure 5d) reveals characteristic peaks corresponding to C 1s (285.6 eV), O 1s (530.8 eV), and N 1s (399.27 eV), along with a distinct signal at 162.9 eV attributable to the S 2p_3_/_2_ orbital of thiol (R-SH) groups [33]. High-resolution spectra (Figure 5e) further confirm the presence of C-S-H structures, indicating the formation of free thiol groups following disulfide bond cleavage [34].

These results provide direct chemical evidence that the hydrogel degradation arises from reduction-induced disulfide bond cleavage. Collectively, the combination of macroscopic degradation behavior and molecular-level characterization establishes a clear structure–mechanism relationship, demonstrating that Gel@Boc-Dc hydrogels possess well-defined and efficient redox-responsive properties.

### 2.5. Drug Loading Performance

**Figure 6:**
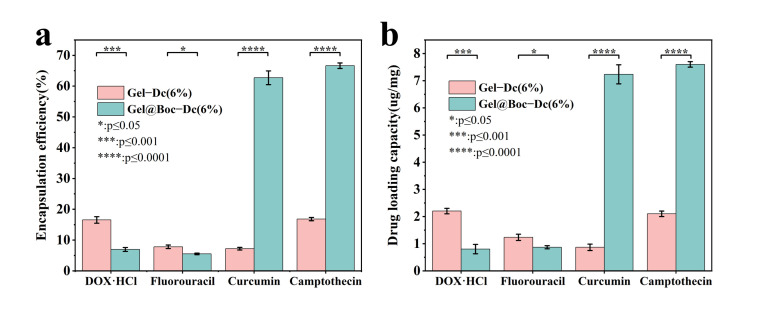
Encapsulation efficiency (**a**) and drug loading capacity (**b**) of native gelatin-based hydrogels (Gel-Dc) and Gel@Boc-Dc toward hydrophilic drugs (doxorubicin hydrochloride and fluorouracil) and hydrophobic drugs (curcumin and camptothecin).

The drug loading performance of Gel-Dc and Gel@Boc-Dc was assessed employing two hydrophilic drugs (doxorubicin hydrochloride and fluorouracil) and two hydrophobic drugs (curcumin and camptothecin) (Figure 6). At a fixed gelatin concentration of 6 wt%, the hydrogels demonstrate substantially different loading behaviors as a function of drug polarity.

For hydrophilic drugs, the encapsulation efficiencies decrease from 16.53% (doxorubicin hydrochloride) and 7.8% (fluorouracil) in Gel-Dc to 6.9% and 5.5% in Gel@Boc-Dc, respectively, with a corresponding reduction in loading content. This decrease reflects the reduced compatibility between hydrophilic drugs and the hydrophobically modified matrix, where Boc groups disrupt favorable polymer–drug interactions [35].

In contrast, Gel@Boc-Dc exhibits significantly enhanced loading capacity for hydrophobic drugs. The encapsulation efficiencies of curcumin and camptothecin reach 62.7% and 66.6%, respectively, considerably higher than those recorded for Gel-Dc (7.2% and 16.8%). This pronounced improvement suggests the presence of strong intermolecular interactions between the modified hydrogel network and hydrophobic drug molecules [36].

**Figure 7:**
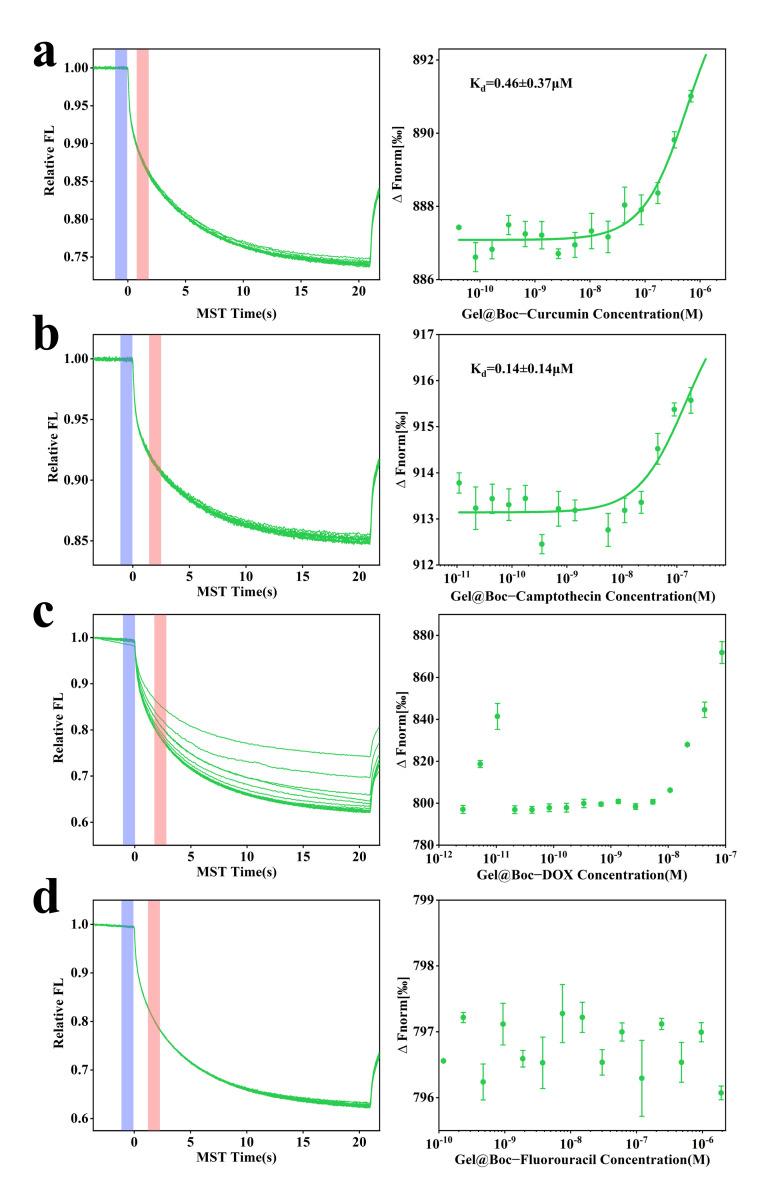
Binding affinity of Gel@Boc toward model drugs determined by microscale thermophoresis (MST): (**a**) curcumin, (**b**) camptothecin, (**c**) doxorubicin hydrochloride, and (**d**) fluorouracil. Gel@Boc exhibited strong binding affinity toward hydrophobic drugs, with dissociation constants (Kd) of 0.46 ± 0.37 µM for curcumin and 0.14 ± 0.14 µM for camptothecin, indicating high-affinity interactions.

To elucidate the origin of this selective loading behavior, the binding affinity between Gel@Boc and the model drugs was further investigated using microscale thermophoresis (MST) (Figure 7). Gel@Boc exhibits strong binding toward hydrophobic drugs, with dissociation constants (K_d_ of 0.46 ± 0.37 µM for curcumin and 0.14 ± 0.14 µM for camptothecin), indicative of high-affinity interactions. In contrast, negligible binding is observed for hydrophilic drugs, as no reliable binding curves can be obtained.

This binding selectivity originates from hydrophobic interactions between the tert-butoxycarbonyl (Boc) moieties and hydrophobic drug molecules, which facilitate stable association in aqueous environments, whereas hydrophilic drugs remain preferentially solvated and thus exhibit minimal interaction with the hydrophobic domains [35].

The strong agreement between MST-derived binding affinity and macroscopic loading performance highlights that drug encapsulation is primarily governed by hydrophobic interactions introduced through Boc functionalization. Collectively, these results demonstrate that Boc modification transforms gelatin into an amphiphilic carrier that selectively enhances the loading of hydrophobic therapeutics.

### 2.6. Stimuli-Responsive Drug Release Behavior

**Figure 8:**
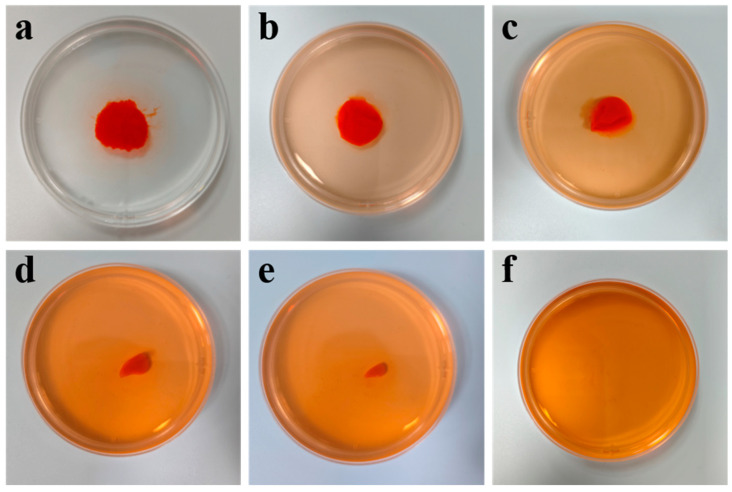
Time-dependent visual observation of doxorubicin hydrochloride release from Gel@Boc-Dc hydrogels in 10 mM DTT solution at (**a**) 5 min, (**b**) 25 min, (**c**) 45 min, (**d**) 65 min, (**e**) 85 min, and (**f**) 105 min, showing progressive hydrogel degradation and drug release.

The reduction-responsive drug release behavior of Gel@Boc-Dc hydrogels was first qualitatively evaluated using doxorubicin hydrochloride-loaded hydrogels (10 mM DTT) (Figure 8). Upon exposure to the reducing medium, the hydrogel undergoes progressive reduction and eventually dissolves, with a gradual increase in the characteristic red color in the surrounding solution, indicating continuous drug release. This behavior reflects the cleavage of disulfide crosslinks, which triggers network disintegration and enables the release of encapsulated drugs.

To quantitatively elucidate the drug release mechanism of Gel@Boc-Dc hydrogels, curcumin was employed as a model hydrophobic drug, and the release profiles were fitted using multiple kinetic models, including zero-order, first-order, Higuchi, and Korsmeyer–Peppas models, to identify the dominant release mechanisms under different environmental conditions.

**Figure 9:**
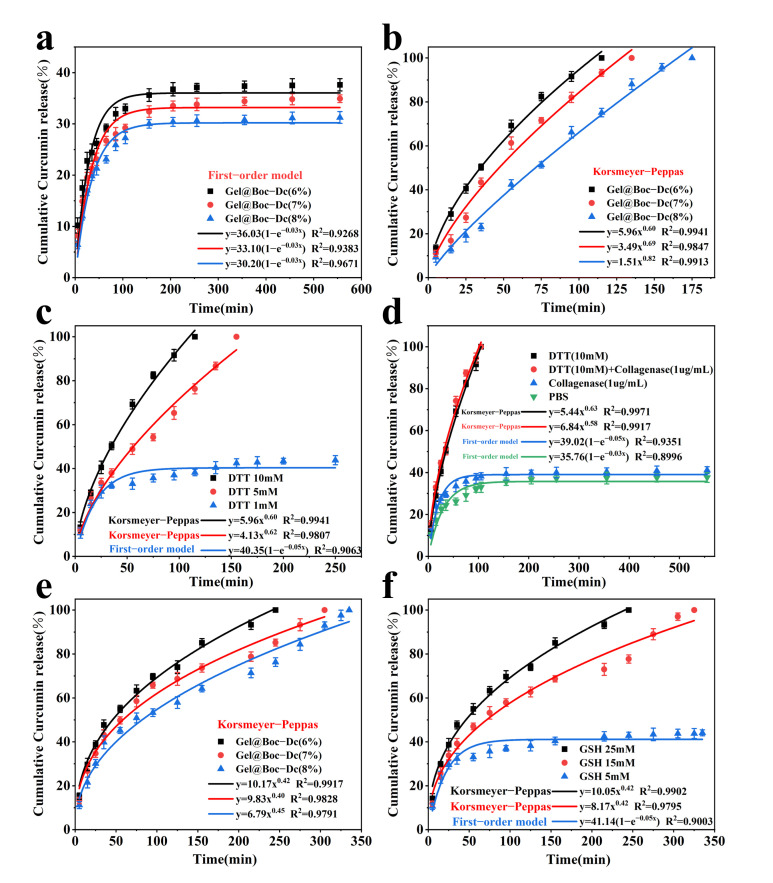
Drug release behavior of Gel@Boc-Dc hydrogels (pH = 7.4, temperature = 37 °C): (**a**) release profiles of curcumin from hydrogels with different gelatin concentrations (6%, 7%, 8%) in PBS; (**b**) release profiles of curcumin from hydrogels with different gelatin concentrations (6%, 7%, 8%) in 10 mM DTT; (**c**) effect of DTT concentration (1, 5, 10 mM) on drug release; (**d**) release profiles of hydrogels with the same gelatin concentration (6%) under four different conditions (PBS, DTT, collagenase, and DTT + collagenase); (**e**) release profiles in GSH solutions with varying gelatin concentrations; (**f**) effect of GSH concentration (5, 15, 25 mM) on drug release.

In PBS (pH 7.4) (Figure 9a), all hydrogels exhibit a characteristic biphasic release profile consisting of an initial burst release followed by a sustained-release phase. The initial stage (0–100 min) is dominated by rapid diffusion of loosely bound or surface-associated drug molecules, whereas the subsequent sustained release is governed by diffusion from the interior of the hydrogel network. The release data in PBS showed the best fit to the first-order model (R^2^ > 0.92), suggesting that drug release under physiological conditions is primarily diffusion-controlled [37]. Moreover, increasing gelatin concentration resulted in a denser network structure with reduced mesh size, thereby restricting molecular diffusion and slightly suppressing overall drug release.

Under reductive conditions (10 mM DTT) (Figure 9b), the release behavior is markedly accelerated due to cleavage of disulfide bonds. In this case, drug release is no longer governed solely by diffusion but is strongly coupled with network degradation. The release profiles exhibited the best fit to the Korsmeyer–Peppas model (R^2^ > 0.98), with diffusion exponent (n) values ranging from 0.60 to 0.82, consistent with a non-Fickian transport mechanism dominated by the combined effects of diffusion and matrix erosion [38,39]. Notably, the n value increased with gelatin concentration (0.60 to 0.82), suggesting progressively enhanced erosion contributions in denser networks [40]. Hydrogels with lower gelatin content (6 wt%) exhibit faster release kinetics, achieving complete release within ≈115 min, whereas those with higher concentrations (8 wt%) require extended time (≈175 min), reflecting the increased structural stability and reduced accessibility of disulfide bonds in denser networks.

The influence of reducing agent concentration was further evaluated (Figure 9c). The release rate increases with DTT concentration, with complete release observed at 10 mM, while only partial release (≈52%) occurs at 1 mM. At high concentrations (10 mM and 5 mM), the release curves exhibited the best fit to the Korsmeyer–Peppas model (R^2^ > 0.98), with n values of 0.6 and 0.62, respectively, confirming the dominant contribution of coupled diffusion and matrix erosion. In contrast, the release profile at 1 mM DTT was better described by the first-order model, indicating that diffusion remained the predominant release mechanism under weakly reducing conditions. These findings demonstrate that the extent and rate of disulfide bond cleavage directly regulate network degradation and, consequently, drug release kinetics.

To more accurately mimic the tumor microenvironment, the contribution of enzymatic degradation was additionally assessed (Figure 9d). In the absence of DTT, collagenase induces a moderate increase in drug release at later stages, indicating gradual enzymatic degradation of the gelatin backbone. In contrast, in the presence of DTT, complete drug release is achieved within 105 min regardless of enzyme addition, demonstrating that the reductive cleavage of disulfide bonds dominates the degradation process under strongly reducing conditions, effectively overshadowing enzymatic contributions.

Given that glutathione (GSH) is the primary intracellular reducing agent, release behavior was further investigated in GSH-containing systems (Figure 9e,f). Although the overall trends are consistent with those observed in DTT, the release kinetics are noticeably slower, requiring longer times to reach completion. Under high GSH concentrations, the release profiles were also well fitted by the Korsmeyer–Peppas model, with n values ranging from 0.40 to 0.45 [41,42]. Compared with DTT-triggered release, the lower n values suggest reduced erosion contributions and enhanced diffusion-controlled release behavior. This milder degradation process prevents abrupt collapse of the hydrogel network and avoids rapid burst release of the encapsulated drug. The slower release kinetics in GSH are likely attributable to its lower reduction potential and slower thiol–disulfide exchange kinetics compared with DTT [43]. Importantly, this behavior suggests that the hydrogel can maintain structural integrity under physiological conditions while enabling sustained and controlled drug release in reductive environments [44].

Collectively, these results demonstrate that drug release from Gel@Boc-Dc hydrogels is governed by a synergistic interplay between diffusion and reduction-triggered network degradation. The kinetic modeling confirms a clear shift from Fickian diffusion in non-reducing conditions to erosion-controlled release in reducing environments, with the transition governed by disulfide bond cleavage. Both polymer concentration and reductive conditions serve as key parameters for modulating release kinetics, providing a tunable platform for the controlled delivery of hydrophobic anticancer drugs.

### 2.7. Biosafety Assessment

**Figure 10:**
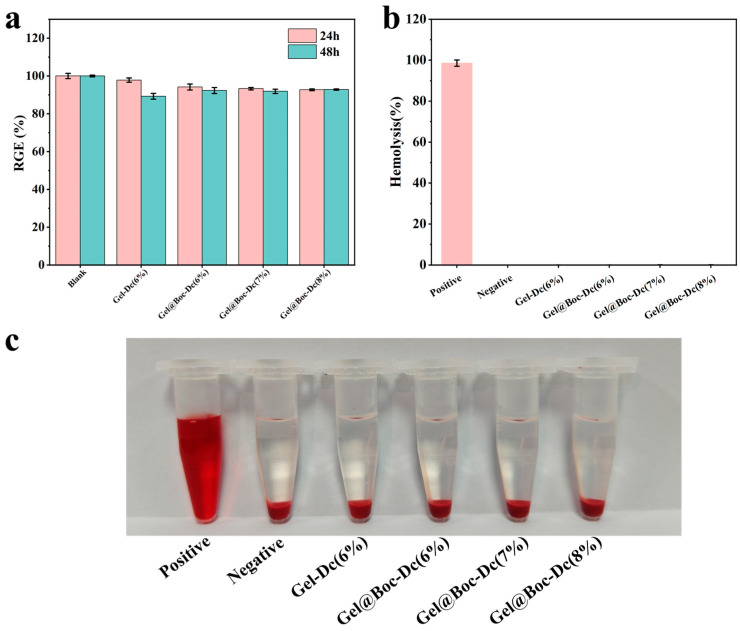
Biocompatibility assessment of Gel-Dc and Gel@Boc-Dc hydrogels. (**a**) Cell viability of HepG2 cells after incubation with hydrogel extracts for 24 and 48 h, evaluated by MTT assay. (**b**) Hemolysis percentage: Quantitative analysis of hemolytic toxicity. All hydrogel experimental groups showed negligible hemolysis (near 0%), well below the 5% safety threshold for biomedical materials. (**c**) Visual results of the samples after centrifugation. The positive control shows a distinct red supernatant due to erythrocyte rupture, while the experimental groups and the negative control exhibit clear supernatants with intact red blood cells settled at the bottom, confirming superior hemocompatibility (data are presented as mean ± SD, n = 3).

For reduction-responsive hydrogels intended for biomedical applications, biocompatibility is a critical prerequisite. The cytocompatibility of Gel@Boc-Dc hydrogels was therefore evaluated using an MTT assay by measuring the relative growth rate (RGR) of HepG2 cells following incubation with hydrogel extracts for 24 and 48 h. As shown in Figure 10a, cell viability across all groups remains approximately 90%, with no significant cytotoxicity observed. These results indicate that neither Boc functionalization nor disulfide crosslinking adversely affects the cytocompatibility of the hydrogel system. Notably, the hydrogel maintains high cell viability while incorporating both hydrophobic modification and reduction-responsive functionality, confirming that the introduced chemical modifications do not compromise biological safety.

Hemolysis, defined as the rupture of red blood cells accompanied by the release of hemoglobin and other intracellular components, is an important indicator for evaluating the blood compatibility of biomaterials. As shown in Figure 10c, all tested hydrogel samples exhibited hemolytic behavior comparable to that of the negative control, indicating negligible membrane-disruptive effects toward red blood cells. Quantitative analysis (Figure 10b) further demonstrated that the hemolysis rates of all hydrogel formulations remained below 1% across the tested concentration range. Collectively, these results confirm the excellent hemocompatibility of the Gel@Boc-Dc hydrogels and suggest their suitability for biomedical applications.

The biosafety of hydrogel degradation products is also an important consideration for their potential biomedical applications. The reduction-triggered degradation of the hydrogel mainly proceeds through the cleavage of disulfide bonds, generating gelatin-derived fragments and cysteine-containing oligomers. Gelatin is a denatured collagen biopolymer whose degradation products are primarily peptides and amino acids that can be further metabolized through normal physiological pathways and are widely recognized as biocompatible and biodegradable biomaterials [45,46]. Moreover, the disulfide cleavage products contain cysteine-related species, which are naturally occurring metabolites involved in glutathione metabolism and cellular redox regulation [47]. Therefore, the degradation products generated from the Gel@Boc-Dc hydrogel are expected to exhibit low biological toxicity and good metabolic compatibility.

## 3. Conclusions

In summary, a reduction-responsive gelatin-based hydrogel with enhanced hydrophobic drug loading capability has been successfully developed through Boc functionalization and disulfide-mediated crosslinking. The introduction of hydrophobic Boc groups transforms gelatin into an amphiphilic matrix, significantly improving its affinity toward hydrophobic drugs, while disulfide bonds confer upon the hydrogel with redox-responsive degradability.

The resulting Gel@Boc-Dc hydrogels exhibit tunable gelation behavior, well-defined network architectures, and markedly enhanced encapsulation efficiency for hydrophobic therapeutics. Under simulated tumor reductive environments, the specific cleavage of disulfide bonds triggers the degradation of the hydrogel network, enabling a transition in drug release kinetics from diffusion-controlled to degradation-controlled. Given its excellent biocompatibility and precise responsiveness, this study provides a reliable strategy for the functionalization of natural biopolymers and offers a highly promising smart platform for targeted anticancer drug delivery.

## 4. Materials and Methods

### 4.1. Materials

Gelatin (Type A, derived from porcine skin) was purchased from Sigma-Aldrich (Darmstadt, Germany). Doxorubicin Hydrochloride (DOX·HCl) was obtained from Shanghai Macklin Biochemical Co., Ltd. (Shanghai, China). Di-tert-butyl dicarbonate, L-Cystine dimethyl ester dihydrochloride, o-Phthalaldehyde (OPA), *N*-Hydroxysuccinimide (NHS), and *N*-(3-Dimethylaminopropyl)-*N′*-ethylcarbodiimide hydrochloride (EDC·HCl) were purchased from Shanghai Aladdin Biochemical Technology Co., Ltd. (Shanghai, China). HepG2 was purchased from Jiangsu KeyGEN BioTECH Co (Jiangsu, China). All other reagents were of analytical grade and used as received without further purification.

### 4.2. Preparation of Boc Grafted Gelation (Gel@Boc)

Gelatin (4.2 g) was dissolved in 50 mL of deionized water under continuous stirring at 50 °C, followed by the addition of 0.5 g sodium bicarbonate to the solution. Separately, di-tert-butyl dicarbonate (1.745 g) was dissolved in 50 mL of tetrahydrofuran (THF). The Boc solution was then added dropwise to the gelatin solution, and the reaction was allowed to proceed at room temperature for 24 h. After completion, THF was removed by rotary evaporation and the residue was dissolved in deionized water. The solution was subsequently dialyzed (MWCO 8–14 kDa, Solarbio, Beijing, China) against deionized water for 48 h, with the external medium replaced every 3–5 h to remove unreacted Boc. The purified product was then lyophilized to obtain Boc grafted gelation.

### 4.3. Preparation of Reduction-Responsive Gelatin-Based Hydrogel (Gel@Boc-Dc)

Lyophilized Gel@Boc (0.21 g) was dissolved in deionized water to prepare solutions with concentrations of 5%, 6%, 7%, and 8% (*w*/*v*). L-cysteine dimethyl ester dihydrochloride (50 mg) was added to each solution and mixed thoroughly. Subsequently, EDC (28.75 mg) and NHS (17.5 mg) were added sequentially, and the mixture was allowed to react at room temperature for 1 h to form hydrogels. The obtained hydrogels were denoted as Gel@Boc-Dc (5%), Gel@Boc-Dc (6%), Gel@Boc-Dc (7%), and Gel@Boc-Dc (8%), respectively.

### 4.4. Characterization of Gel@Boc

#### 4.4.1. ^1^H NMR Analysis

Gelatin and Gel@Boc were dissolved in deuterium oxide (D_2_O) in an NMR tube, and ^1^H NMR spectra were recorded over a chemical shift range of 0–10 ppm on a Nuclear Magnetic Resonance Spectrometer (JEOL JNM-ECZL400S, JEOL, Tokyo, Japan).

#### 4.4.2. Determination of Free Amino Group Content

The free amino content of Gel@Boc was quantified by the o-phthalaldehyde (OPA) method. Lyophilized samples were dissolved in deionized water, and 400 μL of the solution was mixed with 3 mL of fresh OPA reagent. After reacting for 2 min at room temperature, absorbance was measured at 335 nm using a UV-Vis spectrophotometer (NanoPhotometer^®^ NP80, Implen, Munich, Germany). All measurements were performed in triplicate, and serine was used as the standard for calibration. The serine concentration range used for plotting the standard curve is 0–100 μg/mL.

### 4.5. Gelation Behavior of Gel@Boc-Dc Hydrogels

#### 4.5.1. Rheological Measurements

Rheological characterization of the Gel@Boc-Dc hydrogels was conducted on an ARES/G2 rotational rheometer (TA Instruments, New Castle, DE, USA). Dynamic frequency sweeps were performed using a 25 mm parallel plate geometry at 25 °C with a constant strain of 1% over a frequency range of 0.1–100 Hz to obtain the storage modulus (G′) and loss modulus (G″).

#### 4.5.2. Viscosity Testing

The viscosity of the Gel@Boc-Dc hydrogel before and after gelation was determined using a rotational viscometer (DV2T Touch Screen Viscometer, Brookfield, New York, NY, USA). A suitable spindle (e.g., SC4-18 for low-viscosity samples or SC4-34 for higher-viscosity samples) was selected based on the expected viscosity range and fully submerged in the sample. The measurement was conducted at 25.0 ± 0.1 °C via a water bath, with the rotational speed adjusted to maintain a torque reading of approximately 50%. The viscosity was recorded after stabilization, and each test was repeated three times.

#### 4.5.3. Gelation Time

Aliquots of the liquid precursor solutions (prepared in Section 2.2 following the addition of EDC and NHS) were transferred into glass vials and maintained at 25 °C. The vials were inverted at 10 s intervals, and the gelation time was defined as the point at which no flow was observed.

#### 4.5.4. Swelling Performance Test

Lyophilized samples were weighed (*W_d_*) and immersed in 100 mL of PBS buffer (pH 7.4) at room temperature for 24 h. The samples were then removed, gently blotted with filter paper to remove surface water, and weighed again (*W_s_*). Measurements were performed in triplicate, and the equilibrium swelling percentage (ESR) was calculated using the formula below:

(1) $$\mathrm{ESR}\left(\%\right)=\frac{W_{s}-W_{d}}{W_{d}}\times 100\%$$

where *W_d_* and *W_s_* represent the dry and swollen weights, respectively.

### 4.6. Morphological Analysis

#### 4.6.1. Scanning Electron Microscopy (SEM)

The cross-sectional morphology of freeze-dried samples was examined using scanning electron microscopy (EM-30 Plus, COXEM, Daejeon, Republic of Korea) at an accelerating voltage of 20 kV and 931× magnification. Prior to imaging, samples were fractured in liquid nitrogen.

#### 4.6.2. Super-Depth-of-Field Microscope (SDFM)

A Super-Depth-of-Field Microscope (LEICA DVM6A, Leica, Wetzlar, Germany) was employed to examine the micromorphology of the samples. The freeze-dried Gel@Boc-Dc hydrogels were cryofractured in liquid nitrogen, and the resulting flat cross-sections were imaged at 500× magnification.

### 4.7. Reduction-Responsive Analysis

#### 4.7.1. Swelling–Degradation Behavior

Approximately 0.6 g of the Gel-Dc (6%) and Gel@Boc-Dc(6%) hydrogel (recorded as *W*_1_) was immersed in 15 mL of PBS solution (0.02 M, pH 7.4) containing 10 mM DTT, respectively, and incubated at 37 °C for degradation. Every ten minutes, the hydrogel samples were taken out, the surface liquid was blotted with filter paper, and the samples were weighed (recorded as *W*_2_). The relative mass retention percentage (R) was calculated using the following formula:

(2) $$R\left(\%\right)=\frac{W_{2}}{W_{1}}\times 100\%$$

#### 4.7.2. Degradation Behavior

The Gel@Boc-Dc(6%) hydrogels were fully swollen in PBS buffer (pH 7.4). Approximately 0.6 g of the swollen hydrogel (recorded as W_3_) was immersed in 15 mL of PBS solution (0.02 M, pH 7.4) containing 10 mM DTT and in PBS solution without DTT, respectively, and incubated at 37 °C for degradation. Hydrogel samples were collected at 10 min intervals during the initial phase and at 20 min intervals during the subsequent phase. The surface liquid was removed by blotting with filter paper, and the samples were then weighed (recorded as W_4_). The relative mass retention percentage (R) was calculated using the following formula:

(3) $$R\left(\%\right)=\frac{W_{4}}{W_{3}}\times 100\%$$

where W_1_ and W_2_ represent the mass of the sample before and after degradation, respectively.

#### 4.7.3. XPS Analysis

The resulting solution from the degradation experiment was dialyzed (8–14 kDa MWCO) against deionized water for 2 days, with the external buffer replaced every 3–5 h. The dialyzed product was lyophilized, and the surface chemical states were analyzed by X-ray photoelectron spectroscopy (ESCALAB Xi+, Thermo Fisher, Waltham, MA, USA).

### 4.8. Performance Evaluation

#### 4.8.1. Drug Encapsulation and Loading

Freeze-dried hydrogel samples, each with its own actual weight recorded as *W*_5_, were immersed in 10 mL of drug solution (pH 7.4) with an initial concentration of 100 μg/mL (*C*_1_). The mixtures were incubated under continuous stirring at room temperature in the dark for 24 h to allow drug loading equilibrium to be reached. After incubation, the supernatants were collected, and the absorbance at the characteristic maximum absorption wavelength (λ_max_) of each drug was measured using a UV–Vis spectrophotometer (NanoPhotometer^®^ NP80, Implen, Germany). Two hydrophilic drugs, doxorubicin hydrochloride (DOX·HCl, λ_max_ = 480 nm) and 5-fluorouracil (λ_max_ = 266 nm), together with two hydrophobic drugs, curcumin (λ_max_ = 434 nm) and camptothecin (λ_max_ = 370 nm), were employed as model compounds. The residual drug concentration in the supernatant (*C*_2_) was subsequently determined from the corresponding standard calibration curves. The drug loading capacity (LC) and encapsulation efficiency (EE) were calculated according to the following equations:

(4) $$LC\left(\%\right)=\frac{\left(C_{1}{-C}_{2}\right)\times 10}{W_{5}}$$

(5) $$EE\left(\%\right)=\frac{C_{1}{-C}_{2}}{C_{1}}\times 100\%$$

#### 4.8.2. MST Assay for Gel@Boc- and Drug-Binding Assays

Cy5-labeled Gel@Boc was prepared by dissolving Cy5 NHS ester (1.0 mg in 400 µL DMSO) and Gel@Boc (1.0 mg in 400 µL 0.1 M NaHCO_3_, pH 8.0) in a 3 mL glass vial with 15 µL triethylamine, followed by overnight stirring at room temperature in darkness. The labeled product was dissolved in 40% DMSO (for hydrophobic drugs) or aqueous solution (for hydrophilic drugs) and diluted to 400 nM. Stock solutions (curcumin, camptothecin, doxorubicin hydrochloride, and fluorouracil) were prepared in the same solvent system and serially diluted. Each drug dilution was mixed 1:1 (*v*/*v*) with the labeled Gel@Boc solution and analyzed using a Monolith NT.115 instrument (NanoTemper Technologies, Munich, Germany).

#### 4.8.3. In Vitro Drug Release

Drug-loaded hydrogels were placed in 30 mL of release medium and incubated in a shaking water bath (120 rpm, 37 °C). Release media included PBS (pH 7.4), DTT (1, 5, 10 mM), collagenase (1 μg/mL), GSH (5, 15, 25 mM), and their combinations. The effect of gelatin concentration was examined using 10 mM DTT or 25 mM GSH. At predetermined time points, 3 mL of release medium was withdrawn and replaced with fresh medium. The collected samples were stored in the dark at low temperature. Drug release was quantified by UV-Vis spectroscopy and calculated using a standard curve.

(6) $$Releasing\;content\;\left(\%\right)=\frac{amount\;of\;drug\;in\;the\;release\;medium}{amount\;of\;drug\;loaded\;into\;microgels}\times 100\%$$

#### 4.8.4. Cytotoxicity Assay

HepG2 cells were cultured at 37 °C in 5% CO_2_. Lyophilized Gel@Boc-Dc hydrogels were sterilized by UV irradiation (30 min per side). Cells seeded at 6 × 10^3^ cells/well were treated with the hydrogel extract (prepared by incubating 100 mg of hydrogel in 5 mL MEM with 10% FBS at 50 °C). After 24 and 48 h of incubation, the medium was removed, and the wells were washed with PBS. MTT solution (0.5 mg/mL in MEM, 100 μL) was added to each well and incubated for 4 h. The supernatant was discarded, and the formazan crystals were dissolved in DMSO (100 μL). Absorbance was measured at 570 nm using a microplate reader (SPARK 10M, TECAN, Mannedorf, Switzerland).

#### 4.8.5. Hemolytic Toxicity Test

Anticoagulated rabbit blood was centrifuged at 1500 rpm for 10 min. The plasma was removed, and red blood cells (RBCs) were washed repeatedly with prechilled 0.01 M PBS until the supernatant was clear. The washed RBCs were resuspended in the same buffer to prepare a 12% (*v*/*v*) RBC suspension. Aliquots of this suspension were mixed with Gel@Boc-Dc hydrogel extracts of varying concentrations (prepared by incubating 100 mg of hydrogel in 0.01 M PBS at 50 °C) in 1.5 mL tubes. Controls included 0.25% Triton X-100 (positive) and an equal volume of PBS (negative). Each condition was tested in triplicate. The tubes were incubated at 37 °C for 1 h with shaking at 60 rpm, then centrifuged at 1000 rpm for 10 min. Hemolysis was visually assessed: a uniformly transparent red supernatant with no obvious RBC pellet indicated positive hemolysis; a clear, colorless supernatant with an intact RBC pellet indicated no hemolysis. Subsequently, 100 μL of the supernatant was transferred to a 96-well plate, and the absorbance at 540 nm was measured. The hemolysis rate was calculated using the following formula:

(7) $$\mathrm{Hemolysis}\;\left(\%\right)=\frac{A_{sample}-A_{negative}}{A_{positive-A_{negative}}}\times 100\%$$

where *A_sample_*, *A_negative_*, and *A_positive_* are the absorbance (OD) values measured at 540 nm for the sample, negative control, and positive control, respectively.

### 4.9. Statistical Analysis

All experiments were performed in triplicate. Data are expressed as mean ± standard deviation. GraphPad Prism, version 11.0.1 software (GraphPad, San Diego, CA, USA), was used to perform all statistical analyses. Welch’s *t*-test or one-way analysis of variance (ANOVA) was used as appropriate, and differences were considered significant at *p* < 0.05.

## Data Availability

Data will be made available on request.

## Supplementary Materials

The following supporting information can be downloaded at: https://www.mdpi.com/article/10.3390/gels12070614/s1, Figure S1: Free amino acid content in Boc-modified compounds at different ratios.
