# Supplementary figures and images for: Reduction-Responsive Boc-Modified Gelatin-Based Hydrogels for Enhanced Hydrophobic Drug Loading and Controlled Release
Source: Gels. 2026 Jul 9;12(7):614. doi: 10.3390/gels12070614 (PMC13409542; doi:10.3390/gels12070614)

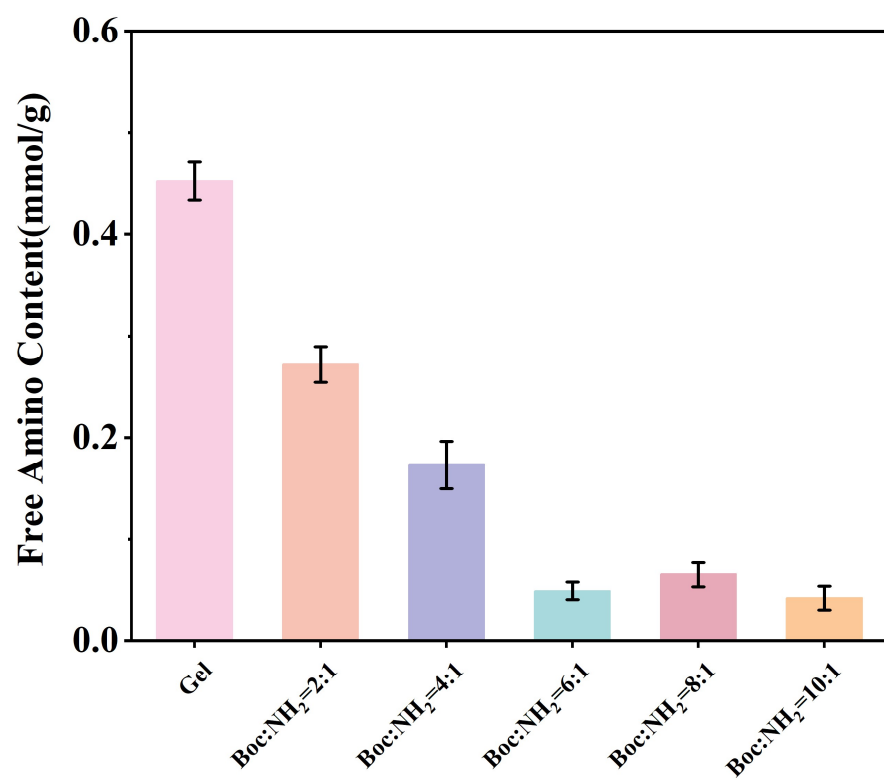

**Figure S1.** Free amino acid content in Boc-modified compounds at different ratios.

Supplement: Supplementary file 1 [file gels-12-00614-s001.zip › Supporting Information.pdf]
